# Cannabis use disorder in relation to socioeconomic factors and psychiatric comorbidity: A cluster analysis of three million individuals born in 1970–2000

**DOI:** 10.1177/14034948221122431

**Published:** 2022-09-18

**Authors:** Rynaz Rabiee, Andreas Lundin, Emilie Agardh, Peter Allebeck, Anna-Karin Danielsson

**Affiliations:** 1 Department of Global Public Health, Karolinska Institutet, Sweden; 2 Centre for Epidemiology and Community Medicine, Sweden

**Keywords:** Cannabis, cannabis use disorder, psychiatric comorbidity, register-based epidemiology, cluster analysis

## Abstract

**Background:**

Cannabis use disorder (CUD) is one of the main reasons for seeking substance use treatment. It is thus important to monitor and increase knowledge of individuals with CUD utilizing healthcare. We aimed to examine the number of CUD diagnoses over time, compare individuals with CUD with those without and identify subgroups based on CUD diagnosis, sex, birth year, socioeconomic factors and psychiatric comorbidity.

**Methods:**

A Swedish, population-based study with 3,307,759 individuals, born in 1970–2000, with register data extending to 2016. K-mode cluster analysis was used to identify potential subgroups.

**Results:**

The number of individuals with a CUD diagnosis was 14,046 (0.42%). CUD diagnoses increased over time (born 1990–1994: 61 per 100,000, born 1995–2000: 107 per 100,000, by 2016). A majority of those with a CUD had another psychiatric diagnosis (80%, compared with 19% for those without CUD). Four clusters were identified. Cluster 1 comprised mainly men with low income and substance use disorders, clusters 2, 3 and 4 comprised mainly women with higher proportions of mood-related, neurotic and stress-related and behavioural disorders.

**Conclusions:**

**There was an increase in CUD diagnoses in Sweden over time, especially among younger birth cohorts. Individuals with CUD were more often male, from younger birth cohorts, with lower education and income than those without CUD. Men and women with CUD exhibited differences in education, income and psychiatric comorbidity. Our results demonstrate the importance of monitoring the impact of socioeconomic factors and psychiatric comorbidity in relation to CUD.**

## Introduction

Cannabis use disorder (CUD) – defined as the *harmful use* of or *dependence* on cannabis – is prevalent, with an estimated 22 million people worldwide meeting the criteria for dependence alone [1]. According to the World Health Organization, 16% of the countries included in their recent ATLAS survey [2] reported cannabis use as the main reason for people to seek substance use treatment, putting cannabis second only to alcohol as a reason for treatment entry. In Europe, the rate of CUD treatment rose between 2010 and 2015 and then plateaued [3]. Moreover, during the last two decades, the demand for cannabis use-related treatment has increased in all Nordic countries [4]. Although cannabis users are a heterogenous group, those in treatment share many of the problems commonly seen in other substance use treatment: multidrug use, co-occurring psychiatric disorders, and social problems. Also, those in treatment are often young and predominantly male [4].

It has been reported that up to roughly one-third of cannabis users may develop CUD [2,5]. A US study showed that CUD was more common among men than women, and among individuals with low income [6]. Importantly, another US study found the transition rate from cannabis use to CUD to be higher among those with psychiatric disorders [5]. Individuals with psychotic disorders and/or personality disorders have been found to be at particularly high risk of transitioning from cannabis use to dependence, with more than half of this group having been reported to develop CUD [5]. Men diagnosed with CUD have been found to exhibit higher rates of other drug use disorders and antisocial personality disorders, whereas women with CUD have been shown to be diagnosed with mood-related and anxiety disorders [6,7]. Furthermore, studies have reported younger age groups (18–25 or 18–29 years) to be at higher risk of CUD than older age groups [8,9].

A recent review on the healthcare utilization of people who use drugs highlighted that studies focusing on cannabis use are lacking, identifying only eight unique study populations and most from the USA [10]. Thus, studies from other countries, and including information on socioeconomic conditions and psychiatric comorbidity, are warranted [11].

Given an overall rise in cannabis use paralleled with an increased potency [12], more persons with CUD in need of healthcare can be expected. Thus, it is important to gain a better understanding and knowledge of the trends in healthcare utilization of individuals with CUD. Additionally, information on specific characteristics of the individuals seeking care is necessary for planning and implementation of appropriate prevention and healthcare measures.

By using national healthcare data, we aimed to study individuals diagnosed with CUD in Sweden between 1990 and 2016. Specifically, we aimed to answer the following research questions:

1. How has the number of CUD diagnoses in healthcare across different age groups developed in Sweden between 1990 and 2016?
2. What characterizes individuals diagnosed with CUD in comparison with individuals without CUD, with regard to sex, birth year, socioeconomic factors and psychiatric comorbidity?
3. To what extent does CUD cluster among individuals with regard to sex, birth year, socioeconomic factors and psychiatric comorbidity?

## Methods

### Study population

Our study population comprised all individuals born in 1970–2000 and registered as living in Sweden some time between 1990 and 2016. Individuals were included in the study population upon their 16th birthday or at the start of the study period (i.e. in 1990), whichever came later. We excluded duplicate health records (*n* = 26), deceased individuals (*n* = 31,794), individuals with missing information on education (*n* = 282,950) or income at year of entry (*n* = 442,218). Our final study population comprised 3,307,759 individuals.

Individuals were identified through the Longitudinal integrated database for health insurance and labour market studies (LISA) [13], which includes socioeconomic variables for all individuals aged 16 years and above, since the year 1990. The study population was linked to the Swedish National Patient Register (NPR), which includes specialized in- and outpatient healthcare [14]. In addition, a subset of the population was linked to the primary healthcare register through the database VAL (Swedish: Vårdanalysdatabaserna, the Stockholm regional healthcare data warehouse), covering all primary healthcare visits in Stockholm region (around 2.2 million inhabitants) [15]. Register linkages were possible through each individual’s unique personal identification number, assigned at birth or migration to Sweden. The study was approved by the Regional Ethics Review Board in Stockholm (Dnr 2010-1185-31-5).

### Main variable

Our main variable was first time of CUD diagnosis as a primary diagnosis in either the NPR or VAL – wherever the CUD diagnosis was recorded first (i.e. unique records) – between 1990 and 2016. We included the following ICD codes: from ICD-9 (utilized until 1996), 3043, that is, cannabis dependence, and from ICD-10 (used from 1997), F12.1 (harmful use) and F12.2 (dependence).

### Covariates

The following variables were obtained from LISA: *Sex. Birth cohort*, based on birth year and categorized into five-year groups. *Disposable family income*, based on all income sources in the family (salaries, wages, welfare benefits, pensions, etc.), for each participant upon their inclusion in the study, categorized into quartiles: low income quartile ⩽SEK234,800, lower-middle quartile SEK234,801–327,400, upper-middle quartile SEK327,401–471,800, high income quartile ⩾SEK471,801 (SEK100 ≈ £10). *Highest attained educational level*, based on number of completed school years and grouped into three categories: primary (⩽ 9 years), secondary (12 years) and post-secondary education (> 12 years).

*Psychiatric comorbidity* included diagnoses that have been found to correlate with cannabis use in previous studies [16]. We chose to include primary and secondary diagnosis as identified in the NPR and VAL. This allowed us to capture individuals with CUD and other psychiatric disorders as well as individuals with psychiatric disorders without CUD, without the groups being mutually exclusive. The included diagnoses were: 1) other substance-related disorders, 2) schizophrenia and other psychotic disorders, 3) mood-related disorders, 4) neurotic and stress-related disorders, 5) personality disorders, and 6) behavioural disorders. The specific ICD codes are detailed in the Supplemental material Table S1 online.

### Statistical analyses

First, we conducted descriptive analyses examining differences between individuals with and without CUD in relation to sex, birth year, socioeconomic factors and psychiatric comorbidity. Second, we employed the k-mode cluster analysis, aiming to explore the composition of characteristics in our sample [17,18]. Clustering is a data-driven method which finds an underlying structure in a dataset by grouping the data points (individuals) based on their similar attributes (variables) [19]. By allowing the data to drive the analysis, we attempted to capture variable combinations in order to find clusters of individuals with similar characteristics. Our dataset comprised only categorical variables, therefore we chose the k-mode clustering method [17,18]. The number of clusters was determined using the elbow method, which plots different numbers of clusters in relation to the cost function. The optimal number of clusters is the point where the slope goes from steep to shallow. Third, we used cross-tabulations to identify cluster compositions. Data management and descriptive analyses were conducted in SAS 9.4. The cluster analysis was conducted in SPYDER 4, a Python software (Python 3.8) available through Anaconda 3.

## Results

### Descriptive results

**Figure 1.**
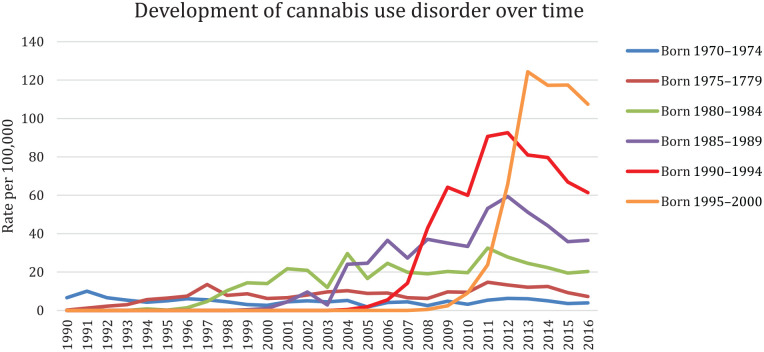
Development of registered cannabis use disorder diagnoses between 1990 and 2016, for each birth cohort respectively.

In the study population of 3,307,759 individuals, 14,046 (0.42%) had a CUD diagnosis. Figure 1 shows the number of CUD diagnoses per 100,000 over time and across birth cohorts.

Individuals with CUD were more often male (78.2%) and belonged to younger birth cohorts than those without CUD (Table I). A majority of those diagnosed with CUD also had another psychiatric diagnosis (80.1%, compared with 19.1% for those without CUD). The highest proportion, 33.6%, was for other substance-related diagnoses, compared with 3.1% in those without CUD.

**Table I.** Distribution of covariate frequencies (%) among individuals with cannabis use disorder (CUD) and without CUD in the entire cohort (*N* = 3,307,759).

		CUD *n* = 14,046	Non-CUD *n* = 3,293,713	Chi-squared
**Sex**	**Men**	10,985 (78.21)	1,680,045 (51.01)	<0.0001
	**Women**	3061 (21.79)	1,613,668 (48.99)	
**Birth cohort**	**Born in 1970–1974**	727 (5.18)	557,402 (16.92)	<0.0001
	**Born in 1975–1979**	1073 (7.64)	495,452 (15.04)	
	**Born in 1980–1984**	1957 (13.93)	490,624 (14.90)	
	**Born in 1985–1989**	2898 (20.63)	558,082 (16.94)	
	**Born in 1990–1994**	4092 (29.13)	614,744 (18.66)	
	**Born in 1995–2000**	3299 (23.49)	577,409 (17.53)	
**Highest attained educational level**	**Primary education, ⩽ 9 years**	6820 (48.55)	540,078 (16.40)	<0.0001
	**Secondary education, 12 years**	6041 (43.01)	1,483,392 (45.04)	
	**Post-secondary education, > 12 years**	1185 (8.44)	1,270,243 (38.57)	
**Income level**	**Low income**, ⩽SEK 234,800	4438 (31.60)	822,851 (24.98)	<0.0001
	**Lower-middle income**, SEK234,801–327,400	3234 (23.02)	824,008 (25.02)	
	**Upper-middle income**, SEK327,401–471,800	3066 (21.83)	823,599 (25.01)	
	**High income** ⩾SEK471,801	3308 (23.55)	823 255 (24.99)	
**Any other psychiatric diagnoses**	**Yes**	11,255 (80.13)	627,672 (19.06)	<0.0001
	**No**	2791 (19.87)	2,666,041 (80.94)	
**Other substance-related disorders**	**Yes**	4720 (33.60)	102,917 (3.12)	<0.0001
	**No**	9326 (66.40)	3,190,796 (96.88)	
**Schizophrenia and other psychotic disorders**	**Yes**	571 (4.07)	13,144 (0.40)	<0.0001
	**No**	13,475 (95.93)	3,280,569 (99.60)	
**Mood-related disorders**	**Yes**	1948 (13.87)	164,354 (4.99)	<0.0001
	**No**	12,098 (86.13)	3,129,359 (95.01)	
**Neurotic and stress-related disorders**	**Yes**	2659 (18.93)	279,033 (8.47)	<0.0001
	**No**	11,387 (81.07)	3,014,680 (91.53)	
**Personality disorders**	**Yes**	352 (2.51)	15,064 (0.46)	<0.0001
	**No**	13,694 (97.49)	3,278,649 (99.54)	
**Behavioural disorders**	**Yes**	2114 (15.05)	99,047 (3.01)	<0.0001
	**No**	11,932 (84.95)	3,194,666 (96.99)	

### Cluster analysis

The elbow method indicated four clusters as ideal (Supplemental material Figure S1).

Cluster 1 was the largest (*n* = 1,524,083), followed by cluster 2 (*n* = 838,483), while clusters 3 and 4 comprised 400,000–500,000 individuals (Table II).

**Table II.** Distribution of covariate frequencies (%) within clusters and comparison between clusters.

		Cluster			
		Cluster 1 *n* = 1,524,083	Cluster 2 *n* = 838,483	Cluster 3 *n* = 509,873	Cluster 4 *n* = 435,320
**Sex**	**Men**	1,187,095 (77.89)	183,744 (21.91)	149,193 (29.26)	170,998 (39.28)
	**Women**	336,988 (22.11)	654,739 (78.09)	360,680 (70.74)	264 322 (60.72)
**Birth cohort**	**Born in 1970–1974**	281,373 (18.46)	199,197 (23.76)	64,823 (12.71)	12,736 (2.93)
	**Born in 1975–1979**	198,480 (13.02)	297,126 (35.44)	0 (0.00)	919 (0.21)
	**Born in 1980–1984**	213,883 (14.03)	161,445 (19.25)	104,603 (20.52)	12,650 (2.91)
	**Born in 1985–1989**	209,036 (13.72)	70,285 (8.38)	276,945 (54.32)	4714 (1.08)
	**Born in 1990–1994**	484,612 (31.80)	90,908 (10.84)	37,906 (7.43)	5410 (1.24)
	**Born in 1995–2000**	136,699 (8.97)	19,522 (2.33)	25,596 (5.02)	398,891 (91.63)
**Highest attained educational level**	**Primary education, ⩽9 years**	136,219 (8.94)	38,588 (4.60)	28,782 (5.64)	343,309 (78.86)
	**Secondary education, 12 years**	1,153,256 (75.67)	156,600 (18.68)	109,435 (21.46)	70,142 (16.11)
	**Post-secondary education, >12 years**	234,608 (15.39)	643,295 (76.72)	371,656 (72.89)	21,869 (5.02)
**Income level**	**Low income**, ⩽SEK234,800	622,880 (40.87)	143,404 (17.10)	31,108 (6.10)	29,897 (6.87)
	**Lower-middle income**, SEK234,900–327,400	290,983 (19.09)	494,245 (58.95)	0 (0.00)	42,014 (9.65)
	**Upper-middle income**, SEK327,600–471,800	290,320 (19.05)	90,188 (10.76)	386,024 (75.71)	60,133 (13.81)
	**High income**, ⩾SEK471,900	319,900 (20.99)	110,646 (13.20)	92,741 (18.19)	303,276 (69.67)
**Any other psychiatric diagnoses**		67,128 (4.40)	18,223 (2.17)	14,076 (2.76)	8210 (1.89)
**Cannabis use disorder**		9575 (0.63)	934 (0.11)	992 (0.19)	2545 (0.58)
**Other substance-related diagnoses**		8079 (0.53)	3368 (0.40)	1496 (0.29)	772 (0.02)
**Schizophrenic and psychotic diagnoses**		8079 (0.53)	3368 (0.40)	1496 (0.29)	772 (0.02)
**Mood-related disorders**		69,603 (4.57)	48,743 (5.81)	29,096 (5.71)	18,860 (4.33)
**Neurotic and stress-related disorders**		117,716 (7.72)	83,936 (10.01)	49,103 (9.63)	30,937 (7.11)
**Personality disorders**		7471 (0.49)	4370 (0.52)	2288 (0.45)	1287 (0.30)
**Behavioural disorders**		50,905 (3.34)	8425 (1.00)	7719 (1.51)	34,112 (7.84)

Cluster 1 consisted mainly of men (77.9%), born in 1990–1994 (31.9%), who had attained secondary education (73.5%) and belonged in the lowest income group (40.9%). The proportion of individuals with CUD was 0.6%. There was a higher proportion of other substance-related disorders (4.4%) than in the other clusters (1.9–2.8%) and a lower proportion of neurotic and stress-related disorders (7.7%) compared with those in clusters 2 or 3 (mean 9.8%). Cluster 1 also comprised a relatively high proportion of individuals with behavioural disorders (3.3%).

Cluster 2 consisted mainly of women (78.1%), born in the 1970s (59.2%), who had attained post-secondary education (76.7%) and belonged in the lower-middle income group (58.5%). This cluster had the lowest proportion of CUD (0.1%) and the highest proportions of mood-related (5.8%) and neurotic and stress-related disorders (10.0%) compared with the other clusters.

Cluster 3 consisted mainly of women (70.7%), born in 1985–1989 (54.3%), who had attained post-secondary education (72.9%) and belonged in the upper-middle income group (75.7%). Cluster 3 included a lower proportion of CUD (0.2%) and a higher proportion of mood-related (5.7%) and neurotic and stress-related (9.6%) disorders.

Cluster 4 consisted mainly of women (60.7%), born in 1995–2000 (91.6%), who had attained primary education (78.9%) and belonged in the highest income group (69.8%). Similar to cluster 1, the proportion of CUD was 0.6%. Cluster 4 had the lowest proportions of all other psychiatric disorders, except behavioural disorders, where it showed the highest proportion (7.8%) compared with the other clusters.

## Discussion

### Main findings

We found that CUD diagnoses increased over time, especially among those born in 1990 and later. About 78% of individuals diagnosed with CUD were male and 80% had an additional psychiatric disorder. Nearly half of the individuals with CUD had attained primary education only, and one-third belonged to the lowest income group, compared with 16% and 24% respectively among individuals without CUD.

We observed a large increase in number of CUD diagnoses in our youngest cohort (born 1995–2000) between the years 2011 and 2016. Their age range during this period was 11–20 years; however, the majority received their first CUD diagnosis between ages 16 and 18 years (not shown). Considering that CUD onset has been shown to occur within the first year of cannabis use, with younger users showing an even higher risk for CUD compared with their older counterparts [20], we would expect an increase in cannabis use approximately during the same time period. The availability of cannabis has increased in Sweden during the past 10 years, although cannabis use has been quite stable during the last 20 years, with self-reported past-month use at about 2–4% among young people (16–29 years) [21]. There has, however, been a slight increase in frequent cannabis use among 16–19-year olds, where those reporting cannabis use have increased their use from an average of four times in 1989 to 13 times in 2016 [22]. Also, an increased cannabis potency in recent years might imply higher rates of harmful effects requiring healthcare. Overall, the increase in CUD diagnoses despite the stability in use is in line with results from a recent Norwegian study [23].

We identified four clusters, where two of the clusters (1 and 4) showed higher proportions of CUD. Cluster 1 included mostly men, a majority born in 1990–1994, in the lower income groups, and with a high proportion of other substance-related diagnoses. Cluster 4 included mostly women, born in 1995–2000, in the highest income group, and with a high proportion of behavioural disorders. The most notable finding, and somewhat in contrast to what we expected, was perhaps the lack of a clear CUD cluster.

Our findings correspond to those of previous studies showing associations between CUD and several psychiatric disorders [5,7,23]. For example, an Australian study showed that seven out of 10 people with a CUD also had another psychiatric disorder [24]. Our findings are also in line with those showing CUD to be more common among men [6] and the younger population (e.g. 18–29 years) [8].

Clusters 1 and 4 had the highest proportions of CUD. The clusters were similar with regard to birth year, educational level and levels of mood-related and neurotic and stress-related disorders. However, they also differed, as cluster 1 included mostly men with low income and cluster 4 included mostly women with high income. Several explanations for their similarities and differences are possible. It may be that the younger population is more sensitive to cannabis use [25], which would put them at a higher risk of developing CUD [8,9]. It may also be that the level of cannabis use is higher in younger age groups [3] and/or that the cannabis used in later years (inevitably relevant for the younger birth cohorts) is more potent with higher concentration of the psychoactive substance ∆-9-tetrahydrocannabinol [25]. Concerning the income differences between these two clusters, some previous studies have shown less affluent adolescents to be at higher risk of frequent cannabis use [26]. High levels of cannabis use during adolescence have also been shown to increase the risk for low income later in life [27]. In contrast, high household income during adolescence has also been associated with high rates of cannabis use [28]. However, none of these studies has considered the possible sex differences with regard to income and CUD, which our study seems to indicate. The similarity in educational level between these two clusters is probably related to the age distribution, especially among those born 1995–2000 who were not old enough to have completed secondary education during our study period.

Clusters 2 and 3 were similar in most regards, including the distribution of sex and psychiatric disorders with high proportions of anxiety and depression. These clusters encompassed the older birth cohorts and essentially all income groups. Official statistics show increased rates of anxiety and depression mainly among women in Sweden during recent years [29]. Interestingly, the composition of cluster 4 was different, with women born in 1995–2000, from affluent circumstances with a higher proportion of CUD and behavioural disorders. Yet, the association between behavioural disorders, such as attention deficit hyperactivity disorder (ADHD), and substance use disorders is well-known and a recent study reported women with ADHD to be almost three times more likely to have a drug use disorder compared with men with ADHD [30]. Still, women may be less likely to be identified as problematic cannabis users within healthcare, since men with CUD to a large extent also exhibit other substance-related disorders, while women instead are diagnosed with mood, neurotic or behavioural disorders. This suggests that more attention should be directed towards women with CUD as they are likely dealing with varied psychiatric disorders.

### Methodological considerations

Our study has some methodological limitations that need to be addressed.

Individuals in our study population, with CUD and any other psychiatric disorder, had sought medical care. Therefore, we measured healthcare utilization for which there may be socioeconomic determinants. Less affluent or vulnerable groups, such as migrants, may be underrepresented due to lower healthcare utilization compared with natives. These factors would likely affect the cluster compositions. Also, our registers do not include individuals who receive cannabis-related care within social services, which in turn may lead to underestimations of the number of individuals with CUD. Thus, the generalization of our results is limited and mainly relevant for contexts with similar healthcare systems and population demographic.

Our definition of CUD was restricted in that the Swedish ICD-9 did not include any code for harmful use of cannabis. This implies that we have identified fewer CUD cases in the years up to 1996, when ICD-10 was implemented. Moreover, the coverage of NPR varies. The inpatient register has complete coverage from 1987, but the outpatient register was included in NPR from 2001 and has a lower coverage of around 80% [14]. As for the VAL database, coverage starts from 2007, and only encompasses a subset of our population [15]. This had implications for the number of cases that we captured during our study period and may have contributed to the increasing trend over time. The improved data quality in later years may be due to better assessment of diagnosis, in addition to better register coverage. Increased knowledge and awareness about psychiatric disorders, including CUD, and decreased stigma may also have increased care-seeking. The low prevalence of CUD in relation to the high comorbidity likely reflects severe cases captured by specialized care, in combination with a long study period enabling registration of several comorbid disorders.

Cluster analyses are well-suited for identifying subgroups in the population based on similar variable attributes and not for studying relationships between variables’ relative importance in association with an outcome. In our study, this enabled identification of subgroups with different characteristics that may influence healthcare needs. A conventional regression analysis would instead have introduced difficulties in assessing the interactions between the psychiatric diagnoses, whereas a cluster analysis circumvents such assessments.

Strengths of this study include the large total population sample with high-quality register data and clinically assessed diagnoses. The use of cluster analyses enabled identification of specific subgroups with different comorbidity and thus potentially different healthcare needs. Discernment of some expected cluster compositions (primarily cluster 1, consisting of men with high proportions of CUD and other substance-related diagnoses) provides some assurance of the validity of the method used. We were able to include a nationwide population, with individuals born over a period of 30 years, and a large number of individuals with a CUD diagnosis (about 14,000).

## Conclusions

There was an increase of CUD diagnoses in Sweden during the study period, especially among younger birth cohorts. Individuals with CUD were more often male, from younger birth cohorts, with lower education and income than those without CUD. Men and women with CUD exhibited differences in education, income and psychiatric comorbidity. Our results demonstrate the importance of monitoring the impact of socioeconomic factors and psychiatric comorbidity in relation to CUD.

## Supplemental Material

sj-docx-1-sjp-10.1177_14034948221122431 – Supplemental material for Cannabis use disorder in relation to socioeconomic factors and psychiatric comorbidity: A cluster analysis of three million individuals born in 1970–2000 Click here for additional data file. Supplemental material, sj-docx-1-sjp-10.1177_14034948221122431 for Cannabis use disorder in relation to socioeconomic factors and psychiatric comorbidity: A cluster analysis of three million individuals born in 1970–2000 by Rynaz Rabiee, Andreas Lundin, Emilie Agardh, Peter Allebeck and Anna-Karin Danielsson in Scandinavian Journal of Public Health
