# Supplementary material for: Cannabis use disorder in relation to socioeconomic factors and psychiatric comorbidity: A cluster analysis of three million individuals born in 1970–2000
Source: Scand J Public Health. 2022 Sep 18;51(1):82–9. doi: 10.1177/14034948221122431 (PMC9903242; doi:10.1177/14034948221122431)

**Supplementary material**

| **Table S1. Overview of ICD-codes in each diagnostic group.** | | | | | | |
| --- | --- | --- | --- | --- | --- | --- |
| **ICD-version** | **Other substance-related disorders** | **Schizophrenia and other psychotic disorders** | **Mood-related disorders** | **Neurotic and stress-related disorders** | **Personality disorders** | **Behavioral disorders** |
| **ICD-10** | F10, F11, F13, F14, F15, F16, F18, F19 | F20, F21, F22 F23, F24, F25 F28, F29 | F30, F31, F32, F33, F34, F38, F39 | F40, F41, F42, F43, F44, F45, F48 | F60, F61, F62, F63, F68, F69, | F90, F91, F92, F93, F94, F98, F99 |
| **ICD-9** | 291, 292, 303, 305 | 293, 295, 296, 297, 298, 299 | 311 | 300, 308, 309 | 301 | 312 |


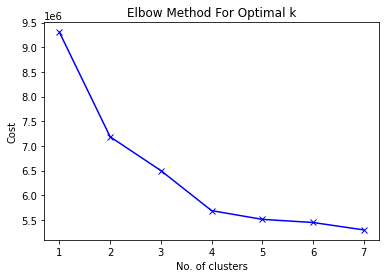

Supplement: sj-docx-1-sjp-10.1177_14034948221122431 – Supplemental material for Cannabis use disorder in relation to socioeconomic factors and psychiatric comorbidity: A cluster analysis of three million individuals born in 1970–2000 [file sj-docx-1-sjp-10.1177_14034948221122431.docx]
